# Adult PKU Clinics in the UK—Users’ Experiences and Perspectives

**DOI:** 10.3390/nu15204352

**Published:** 2023-10-12

**Authors:** Fatma Ilgaz, Suzanne Ford, Michael F. O’Driscoll, Anita MacDonald

**Affiliations:** 1 Department of Nutrition and Dietetics, Faculty of Health Sciences, Hacettepe University, 06100 Ankara, Turkey; 2 Southmead Hospital North Bristol Trust, Bristol BS10 5NB, UK; suzanne.ford@nspku.org; 3 National Society for Phenylketonuria (NSPKU), Sheffield S12 9ET, UK; 4 Department of Adult Child & Midwifery, School of Health Social Care & Education, Middlesex University, London NW4 4BT, UK; m.odriscoll@mdx.ac.uk; 5 Birmingham Children’s Hospital, Steelhouse Lane, Birmingham B4 6NH, UK; anita.macdonald@nhs.net

**Keywords:** Phenylketonuria, PKU, adults, clinic, follow-up, diet, survey

## Abstract

Adults with PKU require life-long management, and ideally, their care should be in a specialised adult metabolic clinic. Their outcomes and co-morbidities have received much attention, but data are lacking on their experience, satisfaction and expectations about the care they receive. This survey reports the experiences and care adults with PKU receive from specialist metabolic clinics in the UK. The online survey developed by the UK NSPKU (National Society for Phenylketonuria), was placed on the NSPKU website from February 2021 to December 2022, and was completed by adults with PKU (≥18 years) or their carers/family members. Sixty-five adult PKU patients and 9 caregivers of adult patients completed the questionnaire (63% female in total). Only 32% of respondents were following a Phe-restricted diet with protein substitute intake as prescribed; the rest were partially adherent or not on dietary restrictions. Nineteen per cent (*n* = 14/74) had not been reviewed in clinic for two years. Half of the respondents (50%) described their experience in adult clinics as “good”. Half of the patients were unable to contact their dietitians with questions or concerns, and only 24% considered that they received adequate support. Clinic reviews usually included anthropometric (82%) and dietary assessments (64%), discussion on management of PKU in daily life (78%) and a blood test (71%). Eighty-eight per cent reported they had at least one neurocognitive, mental health or behavioural co-morbidity but less than half of the patients reported an assessment on their neurocognitive functioning or mental health issues. Adult male patients appeared to have less detailed clinic review than females. Less than half (44%) of the respondents reported that they performed a blood spot for blood Phe at least monthly, but only 32% considered they had been informed about the risk of high Phe levels in adulthood. Although time, cost and stress related to travelling were barriers to a face-to-face review, more than 40% of patients had concerns about remote appointments. The frequency and extent of monitoring of adults with PKU, attending specialist adult services, were less than those specified by the PKU European guidelines. The care of women of reproductive age is prioritised over men. Adult metabolic health services require further attention, development and resources to provide a high standard and equitable service to patients with PKU.

## 1. Introduction

Phenylketonuria (PKU; OMIM 261600) is an autosomal recessive genetic condition characterized by accumulation of the essential amino acid phenylalanine (Phe) in the blood and brain. This is associated with a deficiency of the hepatic Phe hydroxylase (PAH) enzyme, which converts Phe into tyrosine [1,2]. It is a rare disorder with an estimated global prevalence of 1:23,930 live births (range: 1:4500 (Italy)—1:125,000 (Japan)) [3]. Most of the cases are identified through newborn screening [4]. All patients with a diagnostic (pre-treatment) blood Phe level of >360 µmol/L require early and continuous treatment to prevent clinical manifestations of elevated Phe levels, including irreversible brain damage and intellectual disability, microcephaly, seizures, autism, motor deficits, and behavioural problems [5]. Although there are some pharmacological treatment options such as sapropterin dihydrochloride (BH4) and pegvaliase [6,7], a Phe-restricted diet supplemented with a Phe-free or low-Phe protein substitute remains the primary treatment of PKU [8].

Treatment practices have changed throughout the history of dietary management. Prior to the 1980s, a Phe-restrictive diet was stopped before the age of 10 years [9]. However, by 1967, it was noted that elevated blood Phe due to cessation of dietary treatment was associated with declining intelligence quotient and so it was recommended that treatment was continued into early adulthood [10,11,12]. In the 1990s when the first children diagnosed with PKU via newborn screening reached adulthood, a lifelong treatment policy was adopted [13], as a standard of care, aiming to maintain blood Phe within a target therapeutic range for life. Adults with early-treated PKU remain at risk of a wide range of neurological and cognitive problems, particularly if under-treated in adolescence and adulthood [14]. Both historical and concurrent blood Phe control are important [15,16]. It is well established that there is an association between elevated blood Phe levels and neurological symptoms (ataxia, tremor, clumsiness) and impaired cognitive and executive functioning although there is individual variation. This may be associated with the vulnerability of the brain to Phe [17,18,19,20,21,22,23,24,25]. In a review of 39 articles, neurological signs and symptoms were present in adults who were early diagnosed and treated, particularly if they discontinued treatment after childhood [25]. Adults may also have impairments of reasoning, visual–spatial attention, processing speed, sustained attention, visuo-motor control, inhibitory control, working memory and cognitive flexibility [22,26,27] and increased risk of emotional and behaviour deficits (e.g., attention deficit, hyperactivity, depression, anxiety, low mood, mood swings, poor self-image, withdrawal, and a lack of autonomy and drive) [23,28,29]. Psychiatric symptoms such as panic disorders and agoraphobia are reported [30]. Although some of these symptoms may be subtle, they may affect the ability to cope with gaining and maintaining employment, driving, managing money, and family obligations. Adults may lack the self-awareness that any symptoms they experience could be associated with their PKU [17,31]. There are increasing numbers of case reports of adults with PKU who have developed severe neurological manifestations such as spastic paraparesis and loss of visual acuity associated with high blood Phe levels, after long or short periods of treatment discontinuation [21]. There are many gaps in knowledge about the impact of ageing in PKU [32] but there are reports of a higher prevalence of several comorbidities across various organ systems in adults with PKU when compared with age matched non-PKU controls including headaches, eating disorders, hypertension, overweight/obesity, gastritis/esophagitis, osteoporosis, and alopecia [30,33,34].

Lifelong maintenance of low blood Phe levels in adults is necessary. European and American guidelines on the management of PKU recommend maintenance of blood Phe levels in the therapeutic target range of: Europe: 120–360 µmol/L in patients <12 years of age and 120–600 µmol/L in patients ≥12 years; USA: 120–360 µmol/L in all age groups [5,35]. However, blood Phe control deteriorates with age, and most adult patients cannot maintain blood Phe control within target ranges (i.e., blood Phe levels exceeding 600 µmol/L) [29,36,37,38,39]. Some adults may partially follow dietary treatment, commonly without a Phe-free/low-Phe protein substitute. Some may have been advised to stop dietary treatment or even encouraged to make their own decision about the need to follow lifelong therapy [15]. Others may cease dietary treatment as they find it too difficult or impractical to maintain. It is estimated that 23 to 77% of patients stop dietary treatment [24,38,40,41] and their motivation to stay on treatment may be lowered by deficits in executive function and low mood [42]. Over 50% of adults with PKU may be lost to follow-up [25,43]. In Austria, the extent of lost to follow-up was as high as 63%, it increased with advancing age and was higher in men than women [44].

Globally, the number of early treated adults with PKU is increasing and there is a need for consistent lifelong care in a specialised metabolic adult clinic. Management of adults requires a different approach than in childhood, allied to their different medical needs (neuropsychological, psychiatric, reproductive issues, and age-related chronic comorbidities), patient maturation, independence, and social issues. The type and complexity of care changes with time associated with ageing and increasing co-morbidities. To meet adult-specific needs, the European PKU guidelines recommended that all adults be transferred to an adult centre, with a carefully structured transition programme commencing from around the age of 12 years. This may lead to better patient outcomes and higher engagement in clinical care [45,46,47]. Although access to adult clinics is limited throughout Europe [48,49], within the UK there are a network of established adult metabolic centres that provide a service to adults with PKU [41]. Ideally, in adult clinics, there should be access to a (neuro)psychologist, social worker, and neurologist in addition to the core metabolic team [5,25,50]. The PKU European guidelines advise adult patients to have at least monthly blood Phe monitoring and annual outpatient visits including clinical, dietetic and biochemical monitoring to appraise nutritional adequacy, anthropometric assessment, psychological functioning assessment as well as neurocognitive and behavioural screening at age 12 and 18 years in all patients [5].

The issues related to management of PKU in adults have been well-documented particularly from the perspective of the health care professionals working in adult metabolic clinics [40,41,45,48,51]. However, there is lack of information regarding the patient or caregiver experiences and perception of the accessibility, and sufficiency of the medical care they receive in adult hospitals/clinics. This information will enable a better understanding of their medical needs and expectations from the adult clinics, as well as identifying the motivators and barriers of attending follow-up clinics in adulthood. Additionally, it is important to determine if the current monitoring offered in adult clinics meets the recommendations specified by the European PKU guidelines [5].

The National Society for Phenylketonuria (NSPKU) is a charity formed in 1973 to help and support individuals with PKU from all age groups, their parents and families. The NSPKU conducted a survey in 2018 to investigate the practical, social and psychological issues of living with phenylketonuria (PKU) in a group of patients including children and adults [24]. In this current study, the NSPKU primarily aimed to investigate the experiences and expectations of adult patients with PKU or their caregivers in the UK.

## 2. Materials and Methods

### 2.1. Study Questionnaire and Participants

This non-validated cross-sectional study used an online questionnaire to collect data between February 2021 to December 2022. The questionnaire was developed by the UK patient association for PKU (NSPKU) and was aimed at adults with PKU aged 18 years or older with a diagnosis of PKU or the carers or family members of adult patients with PKU aged ≥18 years. The study questionnaire was created on Google Forms, and it was placed on the NSPKU website, and advertised on social media (on the NSPKU Facebook and Twitter accounts). It contained a total of 27 questions, including 22 single or multiple choice, and 5 open-ended questions on demographics, main characteristics of the clinic of PKU care (e.g., type and location of clinic, team members, frequency and context of monitoring and follow-up), characteristics of treatment and related issues (e.g., dietary and medical treatment, adherence, symptoms, and comorbidities) as well as patients’ opinions, expectations, and satisfaction about the clinical care and support they receive.

### 2.2. Data Analysis

Responses to single, multiple-choice, and multiple response questions were summarized as percentages of respondents and were analysed quantitatively using descriptive statistics. Non-parametric inferential statistics were used to test for differences by gender on selected questions. Verbatim reasons for non-attendance to clinic were divided into common themes. Two of the investigators (S.F. and A.M.) selected illustrative quotes from the responses to open-ended questions.

### 2.3. Ethics

Ethical approval was not sought as this was a patient association (NSPKU) survey and respondents were not approached by the National Health Service or educational establishments such as universities. The primary purpose of the survey was clarified at the beginning of the questionnaire, and the respondent’s decision to proceed was taken as informed consent. Potential respondents were also advised that the NSPKU may publish data from the survey in an anonymised form. Adults with PKU and caregivers gave their consent by their voluntary completion and submission of the online questionnaire. If names of individuals or hospitals were mentioned in verbatim abstracts, these were removed.

## 3. Results

### 3.1. Demographics and Type of Dietary Treatment

A total of 74 respondents fully or partially completed the questionnaire. Most of the respondents were adult patients (≥18 years of age) with PKU (88%, *n* = 65/74), and the remaining were the caregiver or a family member of an adult patient with PKU (12%, *n* = 9/74). Approximately two-thirds of respondents were female (63%, *n* = 46/74). The majority of the respondents were living in England (81%), followed by Scotland (10%), Wales (5%), Northern Ireland (3%), or other (1%).

**Figure 1:**
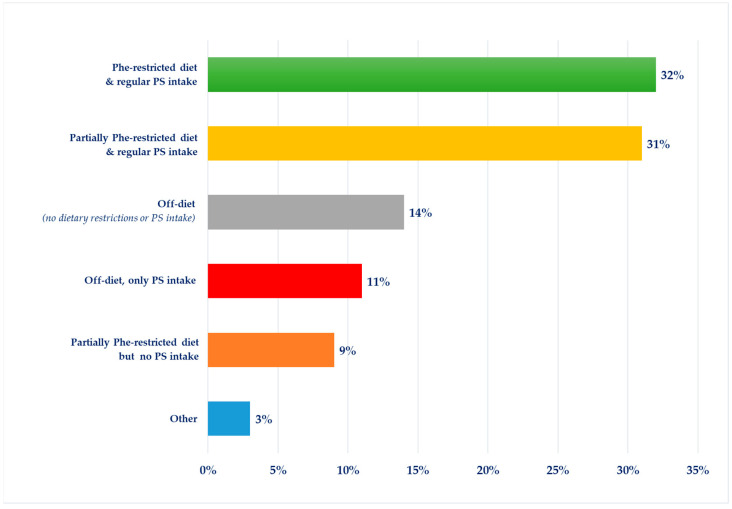
Description of the current diet of adult PKU patients participated in the study (*n* = 74). Abbreviations: Phe, phenylalanine; PS, protein substitute.

Respondents were asked to choose one of six options to describe their diet (or the diet of their patient or the person they care for with PKU) on a typical day, considering the severity of their natural protein/Phe restriction and if they consumed protein substitute regularly. In total, almost two-thirds of the respondents (63%, *n* = 47/74) were either on a Phe-restricted or a partially Phe-restricted diet (which usually avoided most high-protein foods) with regular use of a protein substitute (Figure 1). Nine per cent (*n* = 7/74) of the respondents said they followed a partially Phe-restricted diet but without taking any protein substitute. Twenty-five per cent (*n* = 18/74) of adult patients were off-diet without any restrictions in their protein/Phe intake but nearly half of them (44.4%, *n* = 8/18) were taking a protein substitute. Some patients reported that they were trying to re-establish a Phe-restricted diet but had difficulties taking their protein substitute as prescribed. Some respondents had stopped following their diets due to difficulties and barriers in accessing their special dietary products on prescriptions. One respondent said she “struggled with the diet every day but takes the protein substitute” as she “feels and notices the difference after taking this” (Female, adult PKU patient).

### 3.2. Clinic Attendance

Most of the patients with PKU (74%, *n* = 55/74) attended an adult metabolic clinic where a specialist clinical consultant and dietitian was included in the treatment team. Almost 10% of respondents (*n* = 7/74) reported that there was no metabolic specialist in their clinic; however, 7% (*n* = 5/74) of these respondents said the clinic was supervised by a specialist metabolic team from another hospital.

The majority of the respondents (77%, *n* = 50/65) attended a clinic in the same region of the country where they lived while the remainder (23%, *n* = 15/65) said their clinic was located in a different part of the country (Table 1).

The percentage of patients who attended a face-to-face or a remote (e.g., phone call, video) clinic in the last 12 months was 69% (*n* = 51/74), while in 12% (*n* = 9/74), their last clinic appointment was 1–2 years ago. In the remaining 19% (*n* = 14/74), it had been at least 2 years since their last clinic appointment (Table 1). There was no difference in the responses from male and female respondents.

### 3.3. Barriers to Attending Clinics

Forty-nine of 74 respondents answered the question about the barriers or issues that affected their ability to access hospital care for PKU (Table 1). Thirty-nine per cent of those (*n* = 19/49) indicated that they attended a metabolic clinic, but it was stressful or difficult due to health or mental health issues such as anxiety or coping with the pressures associated with the journey. This was more common in females than in males (43% vs. 29%).

Respondents gave 32 written responses about why they did not attend clinics. They were divided into 5 areas: (1) difficulties obtaining clinic appointments, (2) communication problems, (3) clinic discharge, (4) self-discharge, and (5) denial about PKU. Some identified either that their clinic appointments had been cancelled due to the COVID-19 pandemic, their adult clinic closest to them was still a long distance away; one respondent said: “*The only clinic I can go to is the other side of the country*” (Male, adult PKU patient).

Some respondents experienced anxiety using public transport or felt that it was too expensive to travel to the clinic. Some respondents described inadequate communication and misunderstandings with health professionals about their clinic appointments. Some respondents had been discharged from their clinics during their late adolescence years: “*My son was discharged from a paediatric hospital when he was 16—that was 40 years ago*” (Father of an adult PKU patient). Some respondents had stopped dietary treatment and had not returned to clinic for follow-up: “*I have not been to a PKU hospital since I was in my teens*” (Female, adult PKU patient), or “*I had a baby and went off diet*” (Female, adult PKU patient). Others thought they were managing their condition without support and did not need clinical review: “*My diet seems stable, and I feel I manage my PKU*” (Female, adult PKU patient). One respondent said: “*I didn’t believe I had PKU and just couldn’t accept it*” (Female, adult PKU patient).

Some indicated that they would like to return to clinic but did not have a good experience or did not receive the help they required: “*My mum didn’t believe in doctors and was always negative, so I came off diet completely at about age 13. I finally made the effort to get back in touch with the clinic at the age of 29 years, but I found the support poor*” (Female, adult PKU patient), “*I stopped my diet when I was 14–15 years old, so stopped going to clinic. I tried again in later years, but I did not get much help*” (Female, adult PKU patient).

### 3.4. Monitoring in Clinics

Monitoring in the clinics included anthropometrics measurements (i.e., weight, height; 82%, *n* = 60/73), and discussion of problems related to management of daily living and PKU (78%, *n* = 57/73). Seventy-one per cent (*n* = 52/73) of patients said they had a blood test when attending hospital appointments. Dietary assessment was performed in 64% (*n* = 47/73) of respondents, and nearly half (49%, *n* = 36/73) were able to discuss their wellbeing and quality of life. Medication use was routinely checked in less than half of the respondents (45%, *n* = 33/73). Neurocognitive functioning and psychological/psychiatric problems were only assessed in 27% (*n* = 20/73) and 10% (*n* = 7/73) of respondents, respectively. Only 4 of 73 patients (6%) had an MRI brain scan. There was an evident difference between the percentage of female and male patients having blood tests during appointments; males were less likely to have a blood test at their clinic review (males, 54% vs. females, 80%). In addition, males were less likely to have their height and weight checked (males, 77% vs. females, 84%) and discuss their wellbeing and quality of life (males, 46% vs. females, 53%).

### 3.5. Blood Phe Monitoring

Approximately, half of the respondents (47%, *n* = 30/64) rarely or never performed a blood test at home for monitoring of blood Phe levels, 9% *(n* = 6/64) did blood spots once every few months, and 31% (*n* = 20/64) did a blood spot monthly. The percentage of patients doing a fortnightly or weekly blood spot was only 3% (*n* = 2/64) and 9% (*n* = 6/64), respectively. Female patients were more likely to perform a blood spot for Phe than males (43% of females never or rarely performed blood spots compared to 59% of males). Eighty-three per cent (*n* = 45/74) of respondents typically received their blood results in 1 week or less. 

### 3.6. Clinical, Practical and Social Support

Less than one-third of the respondents (32%, *n* = 23/73) said they were informed by their current clinic about the risks of higher Phe levels in adulthood, and only 15% (*n* = 11) had been advised by their previous clinics. Twenty-one per cent (*n* = 15/72) said that their clinic supports their personal choices about how to manage PKU. Some of the respondents commented that it had been suggested by professionals that they should eat more protein with comments such as “*It wouldn’t matter if I went over on exchanges by up to 5 per day*” (Female patient).

Patients were asked to describe the support they received from their dietitians. A high proportion of the patients were confident that their dietitian had a good understanding of PKU and a Phe-restricted diet (68%, *n* = 50/74) while one female respondent said she was concerned about the adequacy of expertise and experience of PKU her dietitian had. Less than half of the respondents said they could contact their dietitian if they had questions or concerns (49%, *n* = 36/74). The percentage of patients who had enough support from their dietitian was 23% (*n* = 17/74).

Twenty-five per cent of respondents (*n* = 18/72) reported that they obtained treatment for mental health issues from their GP, 7% (*n* = 5/72) from their metabolic clinic (referring to community mental health team) or a metabolic psychologist in the PKU team, and one respondent was referred via their metabolic team to a psychiatrist. Four per cent (*n* = 3/72) paid for mental health treatment privately. Twenty-six per cent (*n* = 19/72) did not want or need help for mental health, as they did not have any issues.

The most common type of practical and social support offered to adults in clinic was “help sorting out problems with getting low protein foods or protein substitutes” (72%, n = 40/55), followed by “help with recipes” (42%, n = 23/55) or “menu planning” (33%, *n* = 18/55), state benefit applications (16%, *n* = 9/55), and shopping lists (15%, *n* = 8/55). Less than 10% of patients received help with education or workplace issues (7%, *n* = 4/55) or had access to a social worker/support (4%, *n* = 2). Males were more likely to seek help in the clinic for applications for state benefits compared to female respondents (32%, *n* = 8 vs. 6%, *n* = 6) (Table 1).

Half of the respondents described their overall experience in the adult clinic as “good” (50%, *n* = 36/72), and said they were “encouraged to discuss the issues” they were experiencing (54%, *n* = 39/72) (Table 1). The clinic experience was described as “relaxed and supportive” and “long enough” by 43% *(n* = 31/72) and 35% (*n* = 25/72), respectively. In contrast, for 14% (*n* = 10/72) of respondents, the duration of the appointment was too short to discuss their problems and concerns, and 10% (*n* = 7/72) reported that clinical appointments were too stressful. However, only 6% (*n* = 4/72) were dissatisfied with their clinical appointments. Female respondents felt less likely that they adequately discussed their problems and concerns compared to males (18%, *n* = 8 vs. 4%, *n* = 1). Some of the respondents said they were disappointed with what they gained from their appointments: *“My daughter often comes away from an appointment feeling despondent, stating ‘nothing new there then*” (Mother of an adult patient with PKU). Some commented that they would like to see a clinical consultant more often as the PKU clinic may be led by other professionals.

More than half of the respondents (58%, *n* = 41/71) reported that they would welcome the opportunity to meet other people with PKU, and almost 70% *(n* = 50/71) would like access to low-protein foods or protein substitute samples during the clinic visits. Some respondents said they needed more help “to tackle the continuous stress of ordering special low protein foods via the GP”. Some thought there should be more help, even rehabilitation, for people who find it difficult to return to dietary treatment. Some felt the PKU teams “should be more proactive and help their patients understand the health issues that might be related to the PKU”. Some requested more information about PKU and impact of ageing.

Respondents were asked to share their thoughts about support from their family, partner or friends (Table 1). Forty-two per cent (*n* = 30/71) of respondents said they would like to have a family member, friend, or partner attend clinic with them. 

### 3.7. Method of Clinic Review

**Figure 2:**
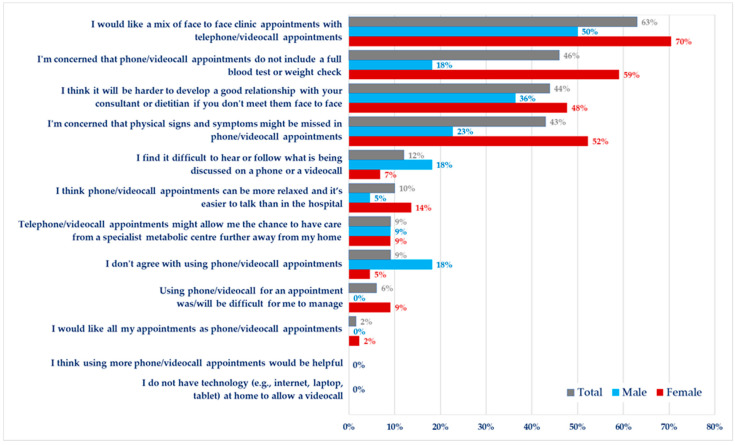
Opinions on the use of technology (e.g., phone/videocalls) for communication in the treatment of PKU (*n* = 68).

Respondents’ opinions about remote consultation/appointments are summarized in Figure 2. Most of the adult females (70%, *n* = 31) and half of the adult males (50%, *n* = 11) stated that they would like a mix of face-to-face and telephone/videocall appointments. However, more than 40% of the respondents expressed their concerns about remote appointments (e.g., phone call/videocall) as they do not include a full blood test or anthropometric assessment (46%, *n* = 31/68), and physical signs or symptoms might be missed in these appointments (43%, *n* = 29/68). Additionally, 44% (*n* = 30/68) of the adult respondents believed that it would be hard to develop a good relationship with their physician or dietitian unless it is a face-to-face appointment in the clinic. A respondent commented: “*I do think you need face-to-face appointments. How else could you look for changes in a patient?*” (Female patient).

However, males appeared to be far less concerned about remote appointments not including physical checks or physical signs (18%, *n* = 4 of males compared to 59%, *n* = 26 of females) or symptoms being missed in video appointments (23%, *n* = 5 of males compared to 52%, *n* = 23 of females). Twelve per cent (*n* = 8/68) said they found it difficult to hear what was being discussed on a phone or videocall. Even though this was more common in males than females (18% vs. 3%), males were likely to support virtual appointments replacing their face-to-face clinic reviews (18%, *n* = 4 males compared to 5%, *n* = 2 females). Respondents stated that with virtual clinics it was “not as easy to have a full or open discussion; there was often multiple attendees, and it felt very distanced.” However, some of the respondents (10%, *n* = 7/68) found it more relaxed than a face-to-face clinic. Only one female respondent said she would like all appointments as phone call/video appointments (Figure 2).

### 3.8. Health Issues: Comorbidities, Psychological, and Behavioural Problems

**Figure 3:**
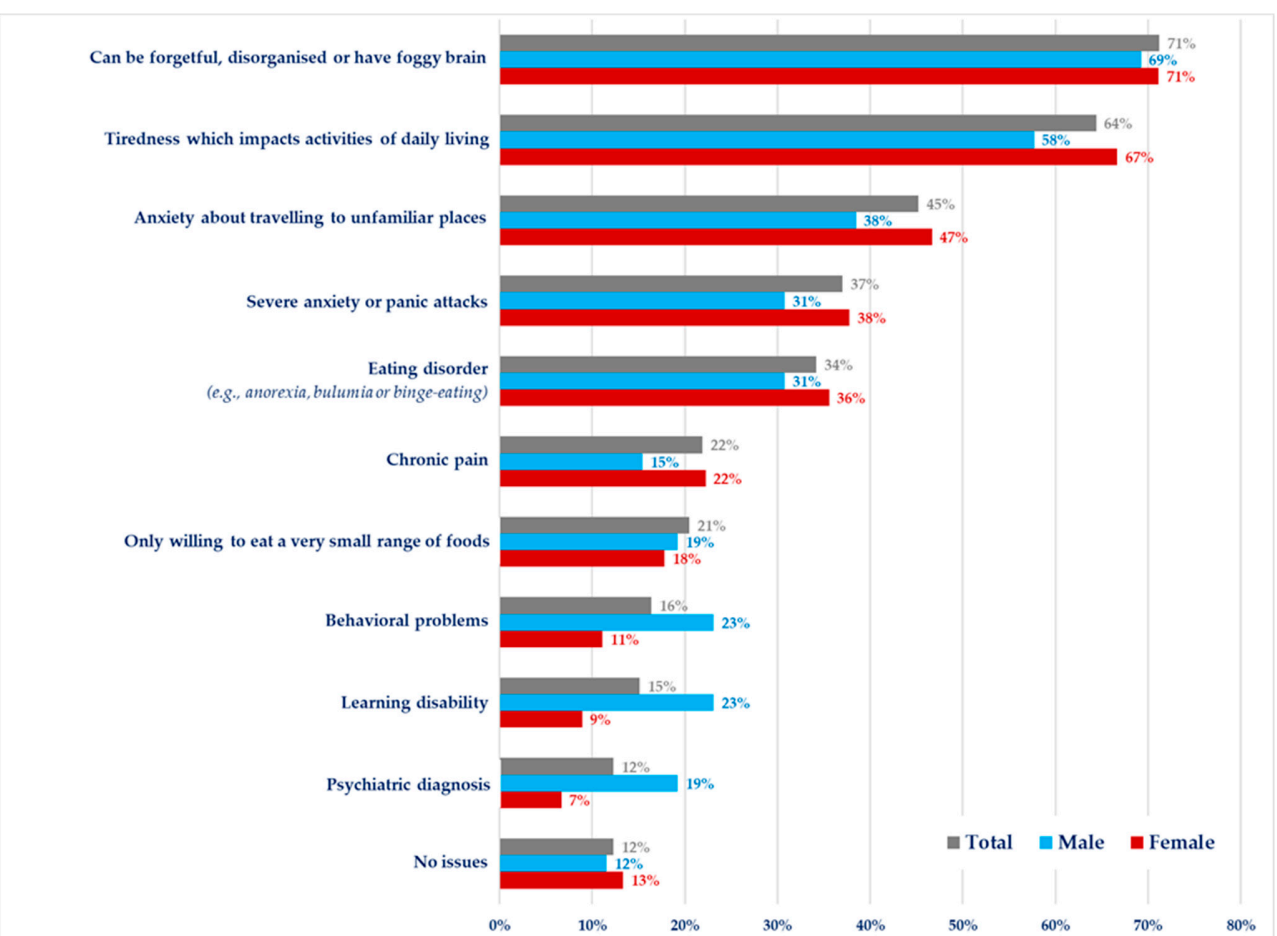
Cognitive, psychological and behavioural problems experienced by adult patients with PKU who participated in the survey (*n* = 73).

Only nine respondents did not report any neurocognitive, psychological or behavioural problems. Of the remaining respondents, many (71%, *n* = 50/73) had problems such as being forgetful, disorganised, or having “foggy” brain, which was similar between female and males (71%, *n* = 32/45 vs. 69%, *n* = 18/26; respectively) (Figure 3). 64% (*n* = 47/73) of respondents said that tiredness impacted on their daily activities. Severe anxiety/panic attacks and anxiety about travelling to unfamiliar places were reported by 37% *(n* = 27/73) and 45% (*n* = 33/73) of respondents, respectively. Thirty-four per cent (*n* = 25/73) self-reported an eating disorder or disordered eating (e.g., anorexia, binge eating, bulimia). Females were more likely to report these health-related issues compared to males. Chronic pain (22%, *n* = 16/73), only being willing to eat a small range of foods (21%, *n* = 15/73), behavioural problems (16%, *n* = 12/73), learning disability (15%, *n* = 11/73), and the presence of a psychiatric disorder (12%, *n* = 9/73) were also reported. The last three problems were reported more by males than females (Figure 3).

**Figure 4:**
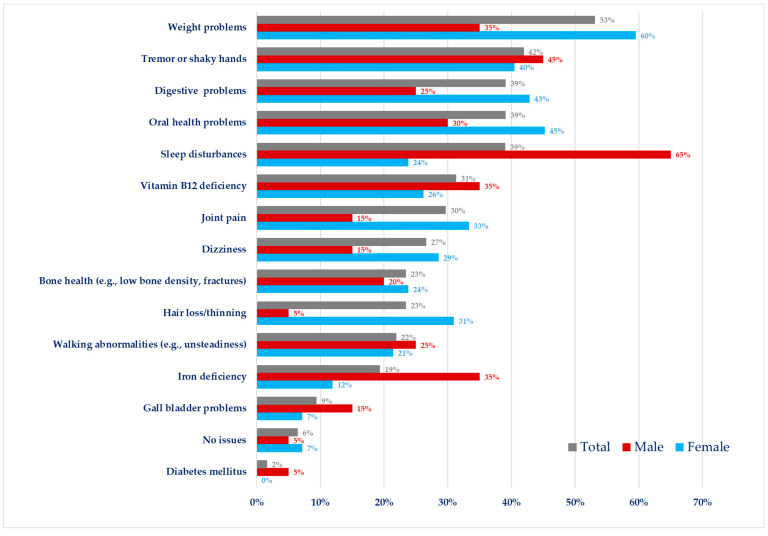
Physical problems or comorbidities of the adult patients with PKU who participated in the survey (*n* = 64).

Respondents were also asked if they (or the person with PKU) had any physical problems or comorbidities that they considered may be related to PKU or the Phe-restricted diet. Weight issues were the most frequent problem reported by 53% of respondents (*n* = 34/64), and females were more concerned about their weight compared to males (60%, *n* = 25/42 vs. 35%, *n* = 7/20). The next most frequent physical problems or comorbidities were tremor/shaky hands (42%, *n* = 27/64), oral health problems (39%, *n* = 25/64), sleep disturbances (39%, *n* = 25/64), and digestive problems (39%, *n* = 25/64). Deficiencies in vitamin B_12_ and iron, joint pain, dizziness, hair loss/thinning, walking abnormalities, and problems related to bone health (e.g., fractures, low bone mineral density) were reported by 20–30% of respondents. Six respondents had gall bladder problems (9%) and one had diabetes (Figure 4).

Weight, oral health, digestive, joint pain, dizziness, hair loss, or hair thinning were more common in females than in males, while males had more complaints of sleep disturbances and vitamin/mineral deficiencies (e.g., iron and vitamin B_12_) compared to females (Figure 4).

## 4. Discussion

The necessity for consistent life-long care from a dedicated specialist PKU team in an adult setting is increasingly recognized [45]. The objective of this NSPKU survey was primarily to determine the experience and satisfaction of adult patients with PKU or their caregivers about the care they received in adult health settings. It aimed to identify “what adult patients with PKU expect from the clinics” and “how much support and care they receive” in comparison to their needs. Information on respondents’ dietary treatment, self-monitoring and monitoring in clinics, self-reported health issues, and opinions on the use of technology (e.g., virtual appointments) was obtained.

It is appropriate that adults with PKU are treated in an adult environment, with teams trained to manage adult issues and comorbidities. Patient care plans should be personalised avoiding under or over treatment, but some core monitoring is essential [52]. The typical staffing in an adult clinic consists of metabolic physician, dietitian, specialist nurse, and possibly a psychologist and social worker. It is important that health professionals build partnerships that will help their patients stay engaged in their care in the long-term. A strong patient–health professional relationship will motivate the patient to remain adherent with the treatment regimen and understand the potential consequences of increased blood Phe levels [52,53,54]. Clinic appointments should be constructive and encouraging as negative feedback may result in failure to attend clinics or clinic transfer. The use of authoritarian strategies may increase feelings of shame, guilt or failure. Continuous support and education are essential [53]. This is particularly important for patients who are struggling to stay on diet or trying to return to their treatment. Overall, only half of the respondents in this survey described their experience in adult clinics as “good”, suggesting that for some patients their expectations were not adequately met. Some respondents complained that the length of their appointments was too short, and for many, the experience was stressful or unsupportive, and even disappointing.

The dietitian is considered an essential member of the core metabolic team, and they play a fundamental role in the successful management of PKU [55]. Since the introduction of national adult PKU clinics, there have been some initiatives to develop training packages for IMD dietitians working with adult patients. In addition, a group of experienced dietitians specializing in IMDs have developed a standard operating procedure outlining the dietetic management of adults with PKU to promote equity of care in IMD dietetic services and to support service provision across the UK [41]. In our study, overall satisfaction about the knowledge, expertise or experience of dietitians was good. However, half of the patients described that they were unable to contact their dietitians with questions or concerns, and only 23% considered that they had adequate support from their dietitians. This might be explained by the limited number of specialized dietitians in the field of IMD, and their heavy workloads. Firman et al. [54] found that knowledge of the PKU diet was associated with dietary adherence, which highlights the importance of ongoing dietetic input in building knowledge and skills for the dietary management of adult patients. Therefore, more specialized dietitians with comprehensive training and expertise in the management of adult PKU are required. The importance of consistency of dietetic support in the maintenance of a Phe-restricted diet for life should be addressed in all dietetic guidelines, and the professional training of metabolic dietitians.

The European PKU guidelines recommended annual outpatient follow-up visits [5] but once- or twice-yearly assessments were recommended by the USA [56]. This survey identified that the rate of clinic attendance (face-to-face or via video link) was high (93%), but only 69% of respondents had been seen within the previous 12 months. Although a study in adolescents and young adults (<21 years) did not show a significant impact of clinic distance on metabolic control, the number of blood Phe samples sent to the clinic was significantly lower in patients residing in distant areas (>100 miles) [57]. Geographic proximity to care was also reported as a major barrier (42%) in a survey conducted in the UK, Netherlands, and Germany [58]. In our study, a considerable number of respondents said that they would prefer a clinic closer to their home to minimise travel, its associated costs or avoid losing workdays [57,59]. It is established that time, transportation challenges, and economic obstacles are an issue associated with clinic visits, and this is particularly enhanced when distance to clinic is increased. It is possible that long-distance healthcare may have an impact on frequency of clinic visits and overall metabolic control in PKU. Almost 25% of respondents were seen in a clinic in a different geographical region to where they lived, but it was unknown if this was because this was the closest PKU clinic or if they had transferred their care to a different clinic.

Although it is known that there are over 1400 adults with PKU in follow-up in English NHS clinics [60], it is unknown how many are lost to follow-up. This survey identified that around 19% of the respondents had not attended a clinic for over two years, which is lower than other studies [44,61]. Reasons given for the loss of follow-up, included travel costs, distance to the metabolic clinic, lack of transportation, fear of travelling, inability to obtain time off work, childcare, lack of perceived benefit from a clinic visit, poor previous experiences, personal choice, or hospital discharge from adult (associated with non-attendance) and paediatric clinics (prior to introduction of lifelong treatment). The financial and time burden associated with dietary treatment have also been reported as major barriers for maintaining dietary treatment [59]. In our survey, some adults felt they had no need to attend clinic as they were self-managing their dietary treatment. Others had negative associations with dietary treatment associated with their earlier experience of PKU care. Health outcomes are likely to be worse for patients without treatment than those in regular follow-up by a metabolic clinic. It is important that all GPs in addition to adult neurology and psychiatry physicians have awareness of PKU in case they review untreated patients with PKU with neuropsychological symptoms [44]. In the UK, there is no single national database listing all patients diagnosed with PKU to monitor the numbers in follow-up care [43].

The National Society for PKU has an important role in helping direct “lost to follow-up” patients to relevant clinics as they may be the first point of patient contact. Any adult who shows an interest in returning to clinic should be seen immediately, as there may be only a small window of opportunity to re-engage them [43]. It is important to modify the clinic approach to suit the individual patient. Flexibility of scheduling appointments and allocating adequate clinic time to discuss all issues is also important. The organisation of satellite clinics so that patients do not have to travel long distances may help. Support (e.g., educational, dietary, and psychological) from the healthcare team has been shown to help patients return to their Phe-restricted diet [62] and is considered as one of the important strategies to successfully re-engage and encourage lifelong management [53]. Organising accessible educational events such as patient webinars, themed workshops, or educational camps may enhance engagement. Education about the disorder or treatment should be tailored to the individual and age-related needs [63]. Regular reminders should be sent prior to clinic to help patients remember appointments. Availability of a social worker/support workers to provide help with financial concerns is an important addition. Issues with prescription costs, inability to access to special low-protein foods or protein substitutes are topics which should be addressed in clinic visits. Even if patients return to clinic, some adult patients may choose to remain off-diet due to the challenging or impractical nature of sustaining a low-Phe diet. These patients should remain in follow-up to monitor their clinical outcomes and nutritional status as well as to keep them informed about new pharmaceutical treatment options [41].

Virtual visits with video clinics may provide an opportunity to complement face-to-face clinic visits, thus ameliorating the impact of geographical barriers and allowing easier access to care [52]. The use of telehealth or remote consultation was common during the COVID-19 pandemic. However, the responses to this survey showed that 40% of respondents expressed their concerns about remote appointments particularly due to the risk of overlooking their symptoms or a shorter review. Females were more interested in a combination of face-to-face and video consultations than males. None of the patients considered that more phone/videocalls would be helpful, and only a small number of respondents found video consultations more relaxed or easier than face-to-face visits. Thus, increasing the number of video consultations may not be the best solution for all adult patients with PKU to increase their attendance at clinic appointments. However, the duration and content of video consultations could be extended to improve patient satisfaction.

Although the frequency of poor outcomes and how they relate to blood Phe in adults has not been fully determined, in our study, the prevalence of the self-reported neurocognitive (e.g., forgetfulness, brain fog), psychological (e.g., severe anxiety, panic attacks) and behavioural problems (e.g., eating disorder/disordered eating) was high (87%). They were more common in females than in males. However, only 25% of patients obtained treatment for mental or psychological health issues via a GP, their metabolic clinic, or a metabolic psychologist in the PKU team and it is possible specialist PKU clinics maybe unaware of the treatment given via the GP. In a previous online survey conducted in three different European countries, the majority of the participants (66%) including adult patients with PKU requested a more extensive annual or bi-annual review and approximately half of them identified psychology evaluation and neuropsychologist assessment as necessary from a speciality clinic [58]. Ideally, psychological support should be integrated into the routine PKU clinic care package. The core PKU team also does not usually include a neurologist, or a psychiatrist and neurocognitive functioning was assessed in only 28% and psychiatric problems were evaluated in only 10% of respondents. In some cases, neurological signs and symptoms can develop slowly over several years upon treatment discontinuation, while in other cases neurological deficits can occur acutely after stopping treatment [25]. The respondents in this study also reported oral health problems, sleep disturbances, digestive problems, joint pain, abnormal walking, and bone problems. Digestive discomfort (bloating and stomach pain), increased use of gastrointestinal agents, sleep issues, and fatigue have been reported by others working with adult patients [28,33,64]. In contrast, although mean bone mineral density is lower in patients with PKU compared to reference groups it is within the normal range in most patients [65] but more evidence is needed regarding elderly patients [66]. We did not collect data on how commonly respondents attended non-PKU clinical appointments for co-morbidities that may relate to PKU.

Interestingly, only one-third of the respondents said they had been informed about the risk of high Phe levels in adulthood, and they may have had an incomplete understanding about the consequences of high blood Phe concentrations. Some adults said they received ambiguous information about the need for strict blood Phe control from health professionals, which caused confusion and potentially lowered motivation. In this survey less than half (44%) of the respondents performed a blood spot for blood Phe monthly or more frequently, despite that treatment guidelines for adult patients recommend monthly (minimum) blood Phe measurements [5,56]. Measurement of blood Phe levels in PKU is essential to evaluate dietary adherence as well as a marker of disorder severity. Patients need to have an understanding that high Phe levels can lead to symptoms that interfere with the ability to adhere to diet and treatment, and that symptoms can be improved when Phe levels are lowered with treatment [21,67,68]. It is established that regular Phe measurements and clinic appointments promote adherence with treatment [39]. There is suggestion that patients who do blood Phe tests infrequently may lower their Phe intake prior to testing and thus have a better blood Phe result, even though this gives an inaccurate reflection of their usual metabolic control [38]. The European PKU guidelines suggest that the time between bloods sampling and patients receiving their blood Phe results should be minimized, aiming for less than 5 days [5]. In this survey, only 35% of the patients received their blood Phe results as recommended, although the majority typically were informed within 1 week. If permitted by health authorities, apps or other technology could help provide more rapid patient feedback about blood Phe concentrations and this should become a standard of care.

In the UK, at the time of survey completion, the only treatment option offered to adult patients with PKU was a Phe-restricted diet. In our study, only 32% of respondents followed a Phe-restriction with protein substitute as prescribed, whilst others either discontinued therapy or followed a partially restricted diet with or without regular protein substitute supplementation. These results are in parallel with the results reported by others [48,54,64]. Partial adherence to dietary treatment or diet discontinuation is usually characterized by insufficient intake or cessation of protein substitute with some natural protein still restricted [39,69]. Any discontinuation of protein substitute, supplemented with vitamins and minerals, increases the risk of micronutrient deficiencies, leading to nutritional deficiencies in protein, calcium, vitamin D, vitamin B_12_, iron, trace elements and decreased bone mineral density. In this survey, respondents described a high frequency of vitamin B_12_, iron deficiency and hair thinning. Both men and women reported a similar frequency of nutritional problems although women reported more hair thinning than men. In another UK study, a group of non-adherent UK adult patients with PKU (*n* = 14) who did not take protein substitute as prescribed, dietary intakes of iron, zinc, vitamin D_3_, magnesium, calcium, selenium, iodine, vitamin C, vitamin A, and copper were significantly lower than adherent patients (*n* = 16) and were below the UK Reference Nutrient Intakes [70]. Rohde et al. [71] demonstrated that in 67 German patients with PKU who consumed ≤0.5 g/kg protein equivalent from a protein substitute that calcium and vitamin D intake was low, and the majority had low plasma 25-OH- vitamin D levels. 

There is evidence that non-adherence to dietary treatment in women may be associated with a higher prevalence of overweight and obesity, which may have implications for increased cardiovascular risk [72,73]. In this survey, as many as 60% of women said they had weight issues. Respondents in our study also indicated a high rate of disordered eating, and this occurred equally in men and women. It has been previously reported that adults with PKU may have food neophobia, disordered eating, and experience less enjoyment of food [74]. Aitkenhead et al. [15] found that partial adherence to PKU diet was also significantly associated with a poorer quality of life compared to those who were on-diet or off-diet. 

Therefore, it is important that adults, particularly those who follow partial dietary treatment have regular monitoring of their BMI and biochemical nutritional status, although in this study, women appeared to receive more nutritional monitoring than men. Women and men should be given equal attention in clinic, with the same number and standard of assessments (e.g., anthropometric measurements, venous blood monitoring), particularly considering the prevalence of partial dietary adherence was similar in both sexes. At a minimum, annual clinical nutritional assessment should be conducted in all adults with PKU, as recommended by the European PKU guidelines. Plasma amino acids, homocysteine and or methylmalonic acid, haemoglobin, MCV, and ferritin, with other nutrients should also be checked [5].

Hence, within clinics, early and regular screening and identification of common symptoms and co-morbidities are essential. Ongoing education and discussion about healthy eating, exercise, dental care, sleep hygiene, any disordered eating, and weight management are important. Brief, validated, easily accessible screening tools that can help monitor symptoms associated with mental health disorders can be utilised in busy clinics [52]. Patient mobile apps may help track any clinical symptoms and assist with monitoring of health and wellbeing. Recommendations for healthcare in adult patients with PKU are summarized in Table 2.

This study has some limitations. Firstly, this study was conducted in the UK and the majority of the respondents were from England; so, the results of this survey may not be a full reflection of care across the UK and may not be generalisable to other countries. The study questionnaire was not validated, and a pilot study was not performed. Data on dietary and medical parameters were obtained based on self-reports. The study was conducted on a convenience sample which mainly consisted of NSPKU members or those who interact with NSPKU on social media. It may not therefore be representative of the general population of people with PKU in England or the UK. There is also a possibility that some of the questions were not fully understood by the respondents. In this survey, there were more female respondents than males. This is not surprising, as adult women are more likely to remain in clinic follow-up due to concerns about the risks of maternal PKU syndrome; hence, they might be more interested in participating in studies. Most of the survey questions were in a multiple-response format, which allows more than one response per respondent. However, this method poses difficulties for inferential statistical tests. Hence, data we analysed were mainly descriptive. We did not collect information about the age of adult patients so some age groups could be under or over represented in this survey. It is possible that the older age group have a higher rate of co-morbidities. We were also unable to compare experience according to different age groups (e.g., younger adults vs. older adults). We also did not ask about the age of onset of any co-morbidities.

## 5. Conclusions

The results of this survey on adult patients living in the UK clearly showed that PKU specialist care for adults requires further attention and development. Self-reported co-morbidities were common and these that could be related to PKU or an effect of partial adherence to treatment. Many respondents did not meet the European PKU guidelines for frequency of follow-up, and almost one fifth were not in regular clinic follow-up. Home blood Phe sampling or monitoring within clinics was limited. Health professionals appeared to prioritize the care of women of reproductive age and were less concerned about health issues of men. Some respondents were dissatisfied with the care they received or would have preferred to follow-up in clinics closer to their homes. Not all the factors that prevent adult patients attending regular clinic follow-ups are easily modifiable. However, continuous support and education, improvement of the quality of healthcare services (e.g., duration of visits, extent of monitoring, number of trained staff) help better engagement with follow-up care. Consistent information about the benefits of treatment adherence and maintaining good metabolic control may achieve better metabolic and clinical outcomes.

## Figures and Tables

**Table 1:** Data on the clinics, monitoring and the experience of adult patients with PKU in clinics.

	Female		Male		Total	
	*n*	%	*n*	%	*n*	%
**Location of the adult clinic (*n* = 65)**						
Same region of residential	33	80.5	16	72.7	50	76.9
Different region	8	19.5	6	27.3	15	23.1
**Time of last appointment (in clinic or remotely) (*n* = 74)**						
<1 year ago	32	69.6	18	69.2	51	68.9
1–2 years ago	6	13.0	3	11.5	9	12.2
2–5 years ago	5	10.9	3	11.5	9	12.2
5–10 years ago	2	4.3	1	3.8	3	4.1
More than 10 years ago	1	2.2	1	3.8	2	2.7
**Issues that affect patients’ ability to receive hospital care for PKU (*n =* 49)** ^a^						
I do not go to the clinic because:						
*… I have health or mental health issues*	1	3.3	2	11.8	3	6.1
*… it does not seem helpful*	2	6.7	2	11.8	4	8.2
*… travelling is too expensive*	1	3.3	3	17.6	4	8.2
*… I cannot take time off work/organise childcare.*	1	3.3	1	5.9	2	4.1
*… I have been discharged/threatened with discharge after missing an appointment*	2	6.7	-	-	2	4.1
I go to a metabolic clinic, but it is stressful or difficult due to health or mental health issues	13	43.3	5	29.4	19	38.8
Other ^b^	16	53.3	8	47.1	25	51.0
	**Female**		**Male**		**Total**	
	***n***	**%**	***n***		***n***	**%**
**Monitoring in face to face clinic visits (*n* = 73)** ^a^						
Dietary assessment (e.g., 3 d food records, 24 h recall)	27	60.0	18	69.2	47	64.4
Anthropometric assessment (weight, height)	38	84.4	20	76.9	60	82.2
Biochemical evaluation (blood tests)	36	80.0	14	53.8	52	71.2
Assessment of neurocognitive functions	12	26.7	8	30.8	20	27.4
Assessment of quality of life and wellbeing	24	53.3	12	46.2	36	49.3
Assessment of psychological/psychiatric problems	5	11.1	2	7.7	7	9.6
Evaluation of problems related to PKU in daily life	36	80.0	19	73.1	57	78.1
Brain scan	3	6.7	1	3.8	4	5.5
Evaluation of medication use	21	46.7	12	46.2	33	45.2
Other	9	20.0	2	7.7	11	15.1
**Overall median satisfaction with the support from the PKU clinic (*n* = 70) ^c^**	44	4.0	24	4.0	70	4.0
**Experience and satisfaction with clinic appointments (*n* = 72)** ^a^						
The overall experience/clinic appointment:						
*… is not good*	3	6.8	1	3.8	4	5.6
*… is good*	24	54.5	12	46.2	36	50.0
*… is stressful*	6	13.6	-	-	7	9.7
*… is relaxed and supportive*	19	43.2	11	42.3	31	43.1
*… is too short*	7	15.9	3	11.5	10	13.9
*… is long enough and not rushed*	16	36.4	9	34.6	25	34.7
*I can’t adequately discuss the problems or concerns I have*	8	18.2	1	3.8	10	13.9
*I am encouraged and supported to discuss the issues I am experiencing*	25	56.8	14	53.8	39	54.2
Other	13	29.5	4	15.4	17	23.6
**Opinions about support from family, friends or others in the clinic (*n* = 71)** ^a^						
I have a brother or sister with PKU and we like to have our clinic visits on the same day	4	9.1	-	-	5	5.6
I like to have my parent/friend/partner attend clinic visits with me	19	43.2	10	40.0	30	42.3
I would welcome the chance to meet other patients with PKU during a clinic visit	24	54.5	15	60.0	41	57.7
I would like the opportunity to get low protein foods or protein substitute samples during clinic visits	31	70.5	17	68.0	50	70.4
Other	6	13.6	2	8.0	8	11.3
	**Female**		**Male**		**Total**	
	***n***	**%**	***n***		***n***	**%**
**Practical and social support offered in the clinic (*n* = 55)** ^a^						
Help with menu planning	11	32.4	6	31.6	18	32.7
Help with making shopping lists	5	14.7	2	10.5	8	14.5
Help with recipes	13	38.2	9	47.4	23	41.8
Help sorting out problems with accessing low-protein foods or protein substitutes	25	73.5	13	68.4	40	72.7
Help with applying for state benefits (e.g., Personal Independence Payment)	2	5.9	6	31.6	9	16.4
Communication to help with education or workplace issues	1	2.9	3	15.8	4	7.3
Access to a social worker/support worker to help with issues we experience	1	2.9	1	5.3	2	3.6
Other	9	26.5	3	15.8	12	21.8

^a^ Multiple-response question. ^b^ Travelling issues (e.g., long distance, not being able to drive), lack of time (e.g., living far from clinic to study at university), lack of an adult PKU clinic in the city they lived, lack of support from their GP to arrange their clinic appointments, and ageing had been reported by the respondents as the reasons for non-attendance. ^c^ There was no statistically significant difference in satisfaction by gender (*p* < 0.05, Mann–Whitney test).

**Table 2:** Recommendations for patient care in adults with PKU.

(1) Adult patients with PKU should be treated in an adult clinic, with a consistent team (including a psychologist and social worker) with all having expert knowledge and skills in adult care and comorbidities.
(2) A transition specific education with participation of both paediatric and adult metabolic teams must be provided to all young patients with PKU before transition to an adult clinic.
(3) Patient care plans should be personalised based on age-related needs; lifestyle (such as treatment access during travel abroad for work or leisure), work patterns, co-morbidities, and ability to manage their own treatment plan. Under- or over treatment should be avoided.
(4) Clinic appointments should be constructive, motivating, and encouraging with the patient considered as an equal partner in their own care. Considering the treatment is self-administered and clinic appointments centre upon adherence to targets, avoidance of “patient shaming”, and positive support is important.
(5) Patients should be given consistent messages about the importance of long-term care and updated about new dietary and pharmaceutical treatment options.
(6) At each clinic visit, individual patient expectations should be established, their knowledge base checked and updated, with detailed screening and investigations of any changes to their physical and mental health status. An annual nutritional assessment should be considered including consideration of disordered eating and measurement of body composition.
(7) Continuous education and support (educational, dietary and psychological) should be provided through patient events such as webinars, themed workshops, or educational camps to enhance engagement.
(8) Support should be given to help patients navigate healthcare, such as access to special low-protein foods, protein substitutes and mental health support.
(9) A social worker/support worker should be a part of the team to discuss and provide help with financial concerns (e.g., prescription costs, clinic transport costs, state benefits).
(10) Virtual clinics should not replace face-to-face clinics. However, the use of technology should be extended to offer an option for some clinic visits, and also to help track clinical symptoms, assist with monitoring of health and wellbeing and provide rapid patient feedback about blood Phe concentrations.
(11) National and international databases should be established of all adult patients with PKU to collect data on co-morbidities and long-term clinical outcome.
(12) All GPs, in addition to adult neurology and psychiatry physicians, should have awareness of PKU.

## Data Availability

Data are contained within the article.
